# The emerging importance of ultradian glucocorticoid rhythms within metabolic pathology

**DOI:** 10.1016/j.ando.2018.03.003

**Published:** 2018-06

**Authors:** Benjamin P. Flynn, Becky L. Conway-Campbell, Stafford L. Lightman

**Affiliations:** Henry Wellcome Laboratories for Integrated Neuroscience and Endocrinology (HW LINE), University of Bristol, Dorothy Hodgkin Building, Whitson Street, Bristol, BS1 3NY, United Kingdom

**Keywords:** Ultradian, Metabolism, Glucocorticoids, Glucocorticoid receptor, RNA polymeraseII, Synthetic steroids, Rythme ultradien, Métabolisme, Glucocorticoïdes, Récepteur des glucocorticoïdes, ARN polymérase II

## Abstract

Glucocorticoid (GC) hormones play significant roles within homeostasis and the chrono-dynamics of their regulatory role has become increasingly recognised within dysregulated GC pathology, particularly with metabolic phenotypes. Within this article, we will discuss the relevance of the ultradian homeostatic rhythm, how its dysregulation effects glucocorticoid receptor and RNA polymeraseII recruitment and may play a significant role within aberrant metabolic action.

## Résumé

Les glucocorticoïdes (GC) jouent un rôle essentiel dans l’homéostasie. La chrono-dynamique de leur rôle régulateur est de plus en plus reconnue dans les pathologies où les GC sont dérégulés, en particulier celles avec un phénotype métabolique. Dans cet article, nous discuterons de la pertinence du rythme homéostatique ultradien, ainsi que les effets de son dysfonctionnement sur le récepteur des glucocorticoïdes, le recrutement de l’ARN polymérase II et de son rôle important dans le développement de syndromes métaboliques.

## 1 Endogenous Glucocorticoid rhythms

**Fig. 1:**
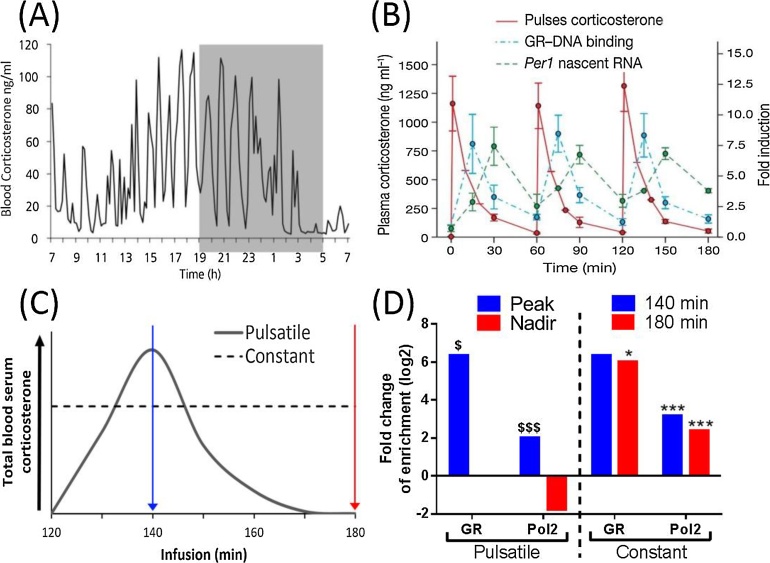
Ultradian glucocorticoid profile exposure directs glucocorticoid receptor binding and modulates RNA polymeraseII recruitment. (A) Blood plasma total corticosterone (cort) levels in rats were sampled every 10 min through jugular cannulae via an automated blood sampling system over 24 hrs (14:10). On average, max amplitudes of hourly pulses varied in a circadian fashion. Modified and re-drawn from Seale, J. V. et al.: Gonadectomy reverses the sexually diergic patterns of circadian and stress-induced hypothalamic-pituitary-adrenal axis activity in male and female rats. J Neuroendocrinol 2004;16:516–24, with permisson. © John Wiley and Sons. (B) Hourly corticosterone (cort) bolus intravenous injections via jugular cannulae into adrenalectomised Sprague Dalwey rats induced rapid and repeatable increases in circulating total cort blood plasma, returning to approximately baseline within 60 min. Resultant GR binding to the gene PER1 GRE and hnRNA production in the liver peaked at 15 min and 30 min respectively, before returning to approximately basal levels by 60 min of each bolus. Plasma cort samples were measured using an enzyme immunoassay. Modified and re-drawn from Stavreva, D. A., et al.: Ultradian hormone stimulation induces glucocorticoid receptor-mediated pulses of gene transcription. Nat Cell Biol 2009;11:1093–102, with permission. © Springer Nature. (C) Sprague Dawley rats were infused hourly with either a pulsatile pattern of cort (20 min infusion followed by 40 min cessation) or a matched constant pattern (infused over 60 min at 0.33 rate) at a dose of 3.84 μM. Liver samples were taken at 140 min or 180 min; corresponding to the peak and nadir of the third pulse. Vehicle infusions were used as control. (D) GR and Pol2 enrichment was detected upstream (∼14.5 kb) and within intragenic regions respectively for the gene TSKU. Pulsatile cort induced time dependent increases at the peak (140 min) of the third pulse for both GR and Pol2 enrichment; Pol2 enrichment in fact was reduced to Vehicle within the nadir (180 min) (adjusted *P*-value $ <0.05, $$$ <0.001). Pattern dependent changes were detected at 180 min for GR whereas both Pol2 time points were significantly increased in response to constant infusion (adjusted *P*-value * <0.05, *** <0.001). Indicating both GR and Pol2 recruitment to the gene TSKU is cort pattern dependent. Liver Chromatin Immunoprecipitation assays with either a GR or Pol2 antibodies were sequenced using the Illumina HiSeq2000 platform and analysed using the Homer suite of tools. Data was analysed to input control and compared to Veh (data un-published).

GC release is regulated by the hypothalamic pituitary adrenal (HPA) axis and plays key roles in circadian, stress, immunological, cognitive and metabolic regulation [1]. Circadian GC zenith is prior to the active period, gradually decreasing to negligible levels at the onset of the inactive period [2]. However, phasic interplay between stimulatory feed-forward and inhibitory feed-back mechanisms within the HPA axis produce naturally oscillating GC pulses; establishing an ‘ultradian’ rhythm that underlies the circadian profile [3], [4], [5]. Fig. 1(A).

## 2 HPA axis dysregulation

Loss of homeostatic GC release can have significant metabolic implications. In Cushing disease cohorts, excessive constant GC production drives significant over-representations of obesity, diabetes mellitus and dyslipidaemia phenotypes (32–41%, 20–47% and 38–71% respectively) [6], [7]. Other instances of continual increases in GC secretion such as three or more stressful events a week, have been shown to increase the likelihood of developing metabolic syndrome phenotypes, ∼2 fold [8]. Also, indications of insulin insensitivity are observed with increased post-prandial glucose and insulin levels more highly induced by bolus hydrocortisone injections at approximatly circadian nadir (17:00) compared to near circadian zenith (05:00) in adrenally suppresed cohorts. Potentially explaining an underlying cause for the ∼1.5 fold higher incidence of metabolic syndrome phenotypes in variable pattern shift workers [9], [10].

## 3 Dynamic glucocorticoid receptor regulation

GCs act via the ligand activated intracellular transcription factor, the glucocorticoid receptor (GR). GR dynamic regulation closely tracks the rising and falling levels in each GC pulse, and may therefore be highly sensitive to altered GC patterns associated with dysregulated GC phenotypes. In vivo studies observed pulsatile GR recruitment to the period circadian clock 1 (PER1) gene's GC response element (GRE) and similar pulsatile nascent RNA production [11]; supporting the potential for ultradian gene transcription within an endogenous system. Fig. 1(B).

## 4 Loss of ultradian GR function

In the brain, the ultradian GC rhythm has been shown to play a critical role in maintaining the physiological, behavioural and molecular response to an acute stress. Replacement of a pulsatile GC rhythm with a matched constant infusion significantly impaired the adaptive response to the stressor [12]. It may therefore be hypothesized loss of, or alterations in the ultradian GC rhythm could also play a pivotal role within metabolism. Notably, synthetic GCs (sGC) inhibit endogenous GC release and replace pulsatile GR activation with prolonged profiles. In vitro experiments demonstrated pulsatile corticosterone treatment induced transient GR:GRE binding (half-life ∼8–9 min), yet binding persisted significantly longer in response to a range of sGC (> 3hr) [11], [13]. In vivo (particularly in the liver) effects may be further prolonged as sGC do not metabolise as rapidly as endogenous GCs [14]. Similar experimental models have shown prolonged constant GR activation induced sustained RNA polymeraseII (Pol2) recruitment compared to a mock ‘ultradian’ profile over a selection of targets [15]. Further, in vivo studies within the rat liver showed gluconeogenic and lipolytic pathways are subject to complex and dynamic alterations in their expression patterns, dependent upon pattern of GC exposure [16] Fig. 1(C-D).

## 5 Conclusion

Ultradian GC action is highly sensitive to disruption and therefore could be equally integral to development of metabolic phenotypes. In support of this, GR and Pol2 recruitment in vitro and in vivo can be differentially modulated depending upon the pattern of cort exposure. New evidence currently emerging suggests that the pattern of delivery, not just the dose, should be considered when treating patients with sGC and that benefits could be garnered from ‘ultradian’ patterned therapies.

## Disclosure of interest

The authors declare that they have no competing interest.
